# 超高效液相色谱-串联质谱法同时测定土壤中30种抗生素

**DOI:** 10.3724/SP.J.1123.2021.02019

**Published:** 2021-08-08

**Authors:** Yu HU, Qingqing ZHU, Ligang HU, Chunyang LIAO

**Affiliations:** 1.中国科学院生态环境研究中心, 环境化学与生态毒理学国家重点实验室, 北京 100085; 1. State Key Laboratory of Environmental Chemistry and Ecotoxicology, Research Center for Eco-Environmental Sciences, Chinese Academy of Sciences, Beijing 100085, China; 2.中国科学院大学资源与环境学院, 北京 100049; 2. College of Resources and Environment, University of Chinese Academy of Sciences (UCAS), Beijing 100049, China; 3.国科大杭州高等研究院环境学院, 浙江 杭州 310000; 3. Institute of Environment and Health, Hangzhou Institute for Advanced Study, UCAS, Hangzhou 310000, China

**Keywords:** 超高效液相色谱-串联质谱, 固相萃取, 抗生素, 土壤, ultra-high performance liquid chromatography-tandem mass spectrometry (UHPLC-MS/MS), solid phase extraction (SPE), antibiotics, soil

## Abstract

土壤基质复杂,土壤中残留的抗生素种类繁多,浓度多为痕量水平,高灵敏度的仪器方法、有效的净化和富集方法、多种类抗生素的同时检测是土壤中抗生素检测的重点和难点。该研究建立了固相萃取-超高效液相色谱-串联质谱法同时测定土壤中7类(磺胺类、氟喹诺酮类、四环素类、大环内酯类、*β*-内酰胺类、酰胺醇类和林可酰胺类)30种抗生素的方法。首先,通过参数优化确定最佳质谱条件,选择BEH-C18色谱柱,以0.1%(v/v)甲酸甲醇溶液-0.1%(v/v)甲酸水溶液为流动相,10%(v/v)甲醇水溶液为进样溶剂。然后,通过提取条件(萃取剂种类及体积)和固相萃取条件(上样液pH、淋洗液有机溶剂比例、洗脱液种类及体积)的优化,确定使用10 mL乙腈和Na_2_EDTA-McIlvaine缓冲液的混合溶液(1:1, v/v)为萃取剂,萃取液pH调节至8.0后,采用HLB小柱进行固相萃取,并以10 mL超纯水淋洗净化,最后用10 mL甲醇-乙腈(1:1, v/v)洗脱目标分析物。在优化的分析条件下,该方法的定量限为0.043~4.04 μg/kg,目标化合物的标准曲线线性关系良好,相关系数在0.992~1.00的范围内,在20、100、200 μg/kg的添加浓度下,大多数目标化合物的加标回收率范围为44.8%~164%,相对标准偏差为0.700%~14.8%。将该方法用于6个实际土壤样品的分析,结果显示在30种抗生素中,17种抗生素有检出,其中12种抗生素的检出率为100%。环丙沙星和诺氟沙星是土壤样品中含量最高的两种抗生素,它们的含量范围分别是13.7~32.1和15.6~43.6 μg/kg。本研究建立的方法简单、快速、溶剂使用量少,能用于土壤样品中痕量水平的7类30种抗生素的同时测定。

## Abstract (en)

The complexity of the soil matrix, as well as the wide spectrum and trace levels of antibiotic residues in soil, make highly sensitive instrumental methods, efficient purification and enrichment methods, and simultaneous determination of multiple antibiotics key and challenging aspects in the analysis of antibiotics in soil. In this study, a solid phase extraction-ultra-high performance liquid chromatography-tandem mass spectrometry (SPE-UHPLC-MS/MS) method was developed for the simultaneous determination of 30 antibiotics (grouped into seven categories: sulfonamides, fluoroquinolones, tetracyclines, macrolides, *β*-lactams, amphenicols, and lincosamides) in soil samples.

In the UHPLC-MS/MS experiment, florfenicol and chloramphenicol were analyzed in the negative ionization multiple-reaction monitoring (MRM) mode, and the other 28 target analytes were analyzed in the positive MRM mode. Sensitive MS conditions were realized by optimizing the instrumental parameters such as collision energy and declustering potential. The effects of the injection solvent (proportion of methanol to water) and mobile phase (types and compositions of the solvents) on the shape and intensity of the chromatographic peaks were studied. The optimized UHPLC conditions were as follows: injection solvent, 10%(v/v) methanol aqueous solution; chromatographic column, BEH-C18 column; mobile phase, methanol and water both containing 0.1%(v/v) formic acid; flow rate, 0.4 mL/min; sample injection volume, 5.0 μL. The effects of the extraction solution (the types and compositions of solvents) and clean-up processes (pH of the loading solution, as well as the types and compositions of the rinse solution and elution solvent) on the method performance were investigated. The acetonitrile/Na_2_EDTA-McIlvaine buffer showed better extraction efficiency for fluoroquinolones than did the methanol/Na_2_EDTA-McIlvaine buffer. Improved recoveries of sulfonamides, macrolides, tetracyclines, and *β*-lactams were observed when the pH of the loading solution was set to 8.0. The recoveries of sulfadiazine and amoxicillin increased with a decrease in the proportion of methanol to water for the rinse solution. Compared to individual methanol or acetonitrile, the methanol-acetonitrile (1:1, v/v) mixture showed better elution efficiency for the target analytes. The optimized pretreatment conditions were determined as follows: the soil sample was spiked with mixed internal standards, and then extracted with 10 mL of acetonitrile/Na_2_EDTA-McIlvaine buffer (1:1, v/v) by shaking for 30 min and ultrasonication for 15 min. The extraction was repeated three times. The sample extract was adjusted to pH 8.0 and loaded onto an Oasis HLB cartridge for purification. The cartridge was rinsed with 10 mL of water to remove impurities and eluted with 10 mL of methanol-acetonitrile (1:1, v/v). Quantitative analysis was conducted using the isotope internal standard method. The method limits of detection and quantification were in the range of 0.013-1.21 and 0.043-4.04 μg/kg, respectively. The correlation coefficients of the calibration curve were 0.992-1.00, suggesting good linearity of the method. At three spiked levels (20, 100, and 200 μg/kg), the average recoveries of most target antibiotics were 44.8% to 164%, and the relative standard deviations were 0.700% to 14.8%. The method was successfully applied to the analysis of the 30 antibiotics in six soil samples. Seventeen antibiotics were detected in the soil samples, and the total contents of the antibiotics in each sample ranged from 73.4 to 184 μg/kg. Twelve antibiotics with a detection frequency of 100% included roxithromycin, clarithromycin, ciprofloxacin, norfloxacin, enrofloxacin, ofloxacin, fleroxacin, lomefloxacin, oxytetracycline, doxycycline, tetracycline, and penicillin G. Ciprofloxacin and norfloxacin were the predominant antibiotics in the soils, with contents in the range of 13.7-32.1 and 15.6-43.6 μg/kg, respectively.

The developed method is simple, rapid, and solvent-saving, and it shows promise for use in the simultaneous determination of trace levels of the 30 antibiotics in soil.

抗生素不仅可以作为药物治疗和预防感染病,还可以作为生长促进剂广泛应用于禽畜养殖业和水产养殖业^[1]^。然而当前抗生素过量使用严重,据统计,每年全球范围内抗生素的使用量在100000至200000吨之间^[2]^。研究表明,人类和动物摄入的抗生素会以母体或活性代谢产物的形式随粪便和尿液排出体外,并在医院、污水处理厂和禽畜养殖场的废水和污泥中富集,最终被排放到水和土壤等环境介质中^[1]^。源源不断进入环境中的抗生素可促进耐药性细菌的增殖,对人类健康造成潜在风险^[3]^。因此,欧盟、美国、澳大利亚和中国均已采取措施限制抗生素的使用,并限定动物肌肉中抗生素的最大残留限量,如欧盟规定动物肌肉中林可霉素的最大残留限量为100 μg/kg^[4]^。《中华人民共和国农业农村部公告第194号》规定自2020年起,停止生产、进口、经营、使用包括抗生素在内的促生长类药物饲料添加剂^[5]^。

建立土壤中多种类多残留抗生素的分析方法可为土壤中抗生素的监测和风险评估提供方法学基础。土壤基质复杂,土壤中抗生素残留种类繁多,且多在痕量水平,高灵敏度的仪器方法、有效的净化和富集方法、多种类抗生素的同时检测是土壤中抗生素检测的重点和难点。抗生素的仪器分析方法包括毛细管电泳法、酶联免疫法、高效液相色谱(HPLC)-紫外/荧光法和高效液相色谱-串联质谱法(MS/MS)等^[6,7,8,9,10,11]^,其中HPLC-MS/MS因其特异性强和灵敏度高等特点,更适合用于土壤中多种类抗生素的痕量分析。土壤中抗生素的净化富集方法包括串联固相萃取、固相萃取(SPE)和QuEChERS(quick, easy, cheap, effective, rugged and safe)等^[10,11,12]^。李晓晶等^[10]^采用SAX-HLB串联固相萃取-超高效液相色谱-MS/MS测定土壤中大环内酯类、氟喹诺酮类和林可酰胺类抗生素,9种目标化合物的定量限为0.80~9.67 μg/kg,加标回收率为65.2%~106%。李兴华等^[11]^利用SPE-高效毛细管电泳法测定土壤中3类(磺胺类、酰胺醇类和*β*-内酰胺类)13种抗生素,定量限为1.33~3.33 μg/kg,加标回收率为78.5%~107%。孟明辉等^[12]^采用QuEChERS-HPLC-MS/MS测定土壤中磺胺类和大环内酯类抗生素,20种目标化合物的定量限为2.0~5.0 μg/kg,加标回收率为61.4%~119%。上述方法定量限低,加标回收率在可接受范围内,但只能同时检测少数几类抗生素。

本研究建立了SPE-UHPLC-MS/MS检测方法,用于土壤中痕量水平的7类(磺胺类、氟喹诺酮类、四环素类、大环内酯类、*β*-内酰胺类、酰胺醇类和林可酰胺类)30种抗生素的同时测定。

## 1 实验方法

### 1.1 仪器、试剂与材料

Exion LC AD超高效液相色谱-串联Triple QUAD^TM^ 5500三重四极杆质谱仪(美国AB Sciex公司); Vortex-Genie 2涡旋振荡器(美国Scientific Industries公司); HS 501 digital摇床(德国IKA公司); 5810 R冷冻离心机(德国Eppendorf公司);氮吹仪(美国Organomation公司); Oasis HLB固相萃取小柱(3 mL,美国Waters公司);固相萃取仪(美国Supelco公司);超纯水仪(美国Millipore公司)。

甲醇和乙腈(色谱纯,美国Thermo Fisher公司);甲酸(分析纯,美国Sigma-Aldrich公司);乙二胺四乙酸二钠(分析纯,上海麦克林生化科技有限公司)、柠檬酸酐(分析纯,阿法埃莎(中国)化学有限公司)、十二水合磷酸氢二钠(分析纯,上海沪试化工有限公司)。

将34.7 g十二水合磷酸氢二钠、18.6 g乙二胺四乙酸二钠和6.5 g柠檬酸酐溶解于500 mL超纯水中,配制得到pH为4.0的Na_2_EDTA-McIlvaine缓冲液。

抗生素标准品:(1)磺胺类(SAs):磺胺嘧啶(SDZ)、磺胺二甲嘧啶(SMZ)、磺胺甲噁唑(SMX)、磺胺甲噻二唑(SMTZ)、磺胺氯哒嗪(SCP)、磺胺对甲氧嘧啶(SFM)、磺胺间甲氧嘧啶(SMM)、磺胺喹噁啉(SQX)、甲氧苄啶(TMP); (2)氟喹诺酮类(FQs):诺氟沙星(NOR)、环丙沙星(CIP)、氧氟沙星(OFL)、洛美沙星(LOM)、恩诺沙星(ENR)、氟罗沙星(FLE)、培氟沙星(PEF)、双氟沙星(DIF); (3)四环素类(TCs):土霉素(OTC)、四环素(TC)、金霉素(CTC)、多西环素(DC); (4)大环内酯类(MLs):克拉霉素(CTM)、罗红霉素(RTM)、脱水红霉素(ETM-H_2_O)、泰乐菌素(TYL); (5)*β*-内酰胺类(BLs):阿莫西林(AMX)、青霉素(PCN-G); (6)酰胺醇类(APHs):氟苯尼考(FF)、氯霉素(CAP); (7)林可酰胺类(LAs):林可霉素(LCM)。磺胺嘧啶、磺胺二甲嘧啶、磺胺甲噁唑、磺胺甲噻二唑、磺胺对甲氧嘧啶、磺胺间甲氧嘧啶、磺胺喹噁啉、甲氧苄啶、诺氟沙星、氧氟沙星、恩诺沙星、克拉霉素、罗红霉素、青霉素、氟苯尼考和氯霉素购自德国DRE公司;磺胺氯哒嗪、洛美沙星、氟罗沙星、培氟沙星、双氟沙星、土霉素、四环素、金霉素、多西环素、脱水红霉素、泰乐菌素、阿莫西林和林可霉素购自加拿大TRC公司;环丙沙星购自中国曼哈格公司;甲氧苄啶-d_3_购自加拿大CDN公司,环丙沙星-d_8_购自德国Witega公司,四环素-d_6_、罗红霉素-d_7_和氯霉素-d_5_购自加拿大TRC公司。

### 1.2 标准溶液的配制

以甲醇为溶剂,将30种抗生素标准品配制成1000 mg/L或100 mg/L的标准储备液,于-20 ℃条件下储存。其中磺胺嘧啶、诺氟沙星、环丙沙星、氧氟沙星、氟罗沙星和培氟沙星需溶解于含0.5%(v/v)的1 mol/L的NaOH溶液的甲醇溶液中,NaOH可与微溶于甲醇的弱酸性物质发生中和反应,促进其溶解。

用甲醇将以上标准储备液稀释配制成4 mg/L的30种抗生素的混合标准溶液,于-20 ℃条件下保存备用。使用前,以10%甲醇水溶液为溶剂,将4 mg/L的30种抗生素的混合标准溶液逐级稀释成200、100、50、20、10、5、2、1、0.5、0.2、0.1、0.05、0.02和0.01 μg/L的系列混合标准溶液。

### 1.3 样品前处理

1.3.1 样品采集

采集表层土壤样品,挑除石子等异物,并进行风干处理,风干后充分研磨和过筛(20目),于-20 ℃条件下保存。

1.3.2 提取和净化

准确称取2.50 g土壤样品,置于15 mL聚丙烯离心管中,加入25 ng抗生素内标混合物(甲氧苄啶-d_3_、环丙沙星-d_8_、四环素-d_6_、罗红霉素-d_7_和氯霉素-d_5_),涡旋混匀。萃取剂为10 mL乙腈和Na_2_EDTA-McIlvaine缓冲液的混合溶液(1∶1, v/v),振荡30 min,超声15 min,提取3次,4 ℃下离心10 min,转速为4000 r/min,移取合并上清液,氮吹浓缩至9 mL,加水稀释至15 mL,调节溶液pH为8.0。

先用6 mL甲醇和6 mL超纯水活化HLB小柱,然后将样品溶液滴加入小柱中。上样后,用10 mL超纯水淋洗,弃去淋洗液,负压抽干20 min,再用10 mL甲醇-乙腈(1∶1, v/v)溶液洗脱。洗脱液氮吹浓缩至干,再用10%(v/v)甲醇水溶液复溶至1 mL,待UHPLC-MS/MS分析。

### 1.4 分析条件

色谱柱:ACQUITY BEH-C18色谱柱(100 mm×2.1 mm, 1.7 μm;美国Waters公司),配有预柱ACQUITY BEH-C18色谱柱(5 mm×2.1 mm, 1.7 μm;美国Waters公司);柱温:40 ℃;流动相:A相为0.1%(v/v)甲酸水溶液,B相为0.1%(v/v)甲酸甲醇溶液;流速:0.4 mL/min。正离子模式下流动相洗脱梯度:0~2.5 min, 10%B~15%B; 2.5~5.5 min, 15%B~60%B; 5.5~6.0 min, 60%B~95%B; 6.0~8.0 min, 95%B; 8.0~8.1 min, 95%B~10%B; 8.1~9.0 min, 10%B。负离子模式下流动相洗脱梯度:0~1.0 min, 10%B~15%B; 1.0~3.0 min, 15%B~60%B; 3.0~4.0 min, 60%B; 4.0~4.5 min, 60%B~95%B; 4.5~6.0 min, 95%B; 6.0~6.1 min, 95%B~10%B; 6.1~7.0 min, 10%B。

离子源:电喷雾电离源(ESI);多反应离子检测模式(MRM)。正离子模式下,离子源温度500 ℃,离子喷雾电压5500 V,气帘气压力35.0 kPa,碰撞气压力7 kPa,喷雾气压力40.0 kPa,辅助加热气压力60.0 kPa。负离子模式下,离子源温度550 ℃,离子喷雾电压-4500 V,气帘气压力40.0 kPa,碰撞气压力9 kPa,喷雾气压力50.0 kPa,辅助加热气压力50.0 kPa。30种抗生素的其他质谱参数见表1。

**表1:** 30种抗生素的质谱分析参数

Compound	Ion mode	Precursor ion (*m/z*)	Product ion (*m/z*)	CE/ eV	DP/ V	Compound	Ion mode	Precursor ion (*m/z*)	Product ion (*m/z*)	CE/ eV	DP/ V
Sulfamonomethoxine	ESI^+^	281.2	156.2^*^	25	90	Lomefloxacin	ESI^+^	352.1	308.4^*^	25	20
(SMM)			92.2	40	90	(LOM)			265.3	30	20
Sulfamethoxazole	ESI^+^	254.2	156.2^*^	23	20	Fleroxacin	ESI^+^	370.2	326.3^*^	25	80
(SMX)			92.2	35	20	(FLE)			269.1	35	80
Sulfaquinoxaline	ESI^+^	301.2	156.1^*^	24	100	Roxithromycin	ESI^+^	837.7	679.5^*^	30	20
(SQX)			92.2	38	100	(RTM)			158.3	42	20
Sulfameter	ESI^+^	281.3	156.2^*^	25	40	Clarithromycin	ESI^+^	748.7	590.4^*^	25	60
(SFM)			92.2	40	40	(CTM)			158.3	35	60
Trimethoprim	ESI^+^	291.3	230.3^*^	32	10	Erythromycin-H_2_O	ESI^+^	716.6	558.5^*^	35	40
(TMP)			123.3	30	10	(ETM-H_2_O)			158.3	33	40
Sulfamethazine	ESI^+^	279.2	186.3^*^	24	80	Tylosin	ESI^+^	916.7	772.6^*^	40	20
(SMZ)			156.2	26	80	(TYL)			174.3	45	20
Sulfadiazine	ESI^+^	251.2	156.2^*^	20	20	Oxytetracycline	ESI^+^	461.3	443.2	22	60
(SDZ)			92.2	30	20	(OTC)			426.3^*^	27	60
Sulfamethizole	ESI^+^	279.2	156.3^*^	20	17	Chlortetracycline	ESI^+^	479.3	462.3	25	40
(SMTZ)			92.3	35	17	(CTC)			444.2^*^	30	40
Sulfachlorpyridazine	ESI^+^	285.2	156.2^*^	20	20	Doxycycline	ESI^+^	445.3	428.3	25	40
(SCP)			108.2	30	20	(DC)			410.3^*^	27	40
Ciprofloxacin	ESI^+^	332.3	314.4^*^	30	60	Tetracycline	ESI^+^	445.3	427.2	20	40
(CIP)			288.4	25	60	(TC)			410.2^*^	30	35
Norfloxacin	ESI^+^	320.3	302.4^*^	30	60	Amoxicillin	ESI^+^	366.2	349.3^*^	12	35
(NOR)			276.3	25	60	(AMX)			114.3	30	20
Ofloxacin	ESI^+^	362.3	318.4	37	10	Penicillin-G	ESI^+^	335.2	217.3^*^	20	190
(OFL)			261.3^*^	27	10	(PCN-G)			202.2	47	130
Enrofloxacin	ESI^+^	360.4	316.4^*^	25	20	Lincomycin	ESI^+^	407.2	359.4^*^	27	20
(ENR)			245.3	35	20	(LCM)			126.3	35	20
Difloxacin	ESI^+^	400.3	382.4^*^	30	40	Florfenicol	ESI^-^	355.6	185.1^*^	-25	-80
(DIF)			356.4	27	40	(FF)			119.1	-45	-80
Pefloxacin	ESI^+^	334.3	316.3^*^	30	40	Chloramphenicol	ESI^-^	321.3	257.0^*^	-15	-80
(PEF)			290.3	25	40	(CAP)			152.2	-22	-80

* Quantitative ion; CE: collision energy; DP: declustering potential.

## 2 结果与讨论

### 2.1 分析条件的优化

2.1.1 质谱条件的优化

根据7类抗生素的分子结构特征,并参考相关文献^[13,14]^,酰胺醇类的氟苯尼考和氯霉素在ESI^-^模式下进行质谱分析,其他28种抗生素均在ESI^+^模式下进行质谱分析。采用针泵注射器,将30种100 μg/L的标准溶液分别注入离子源,流速为7 μL/min。

对单个抗生素进行质谱条件优化,首先进行全扫描和子离子扫描,参考化合物信息,选取合适的母离子和子离子作为目标化合物的定量、定性离子对,然后在MRM模式下,对目标化合物的定量、定性离子对进行CE、DP等质谱参数的优化。最终30种抗生素的质谱优化参数见表1。

2.1.2 色谱条件的优化

由于7类抗生素的分子结构差异较大,本实验选择了具有广谱性和化学稳定性的BEH-C18色谱柱。比较了分别以甲醇-水、甲醇-0.1%(v/v)甲酸水溶液、甲醇-0.2%(v/v)甲酸水溶液、甲醇-含2 mmol/L醋酸铵的0.2%(v/v)甲酸水溶液、0.1%(v/v)甲酸甲醇溶液-0.1%(v/v)甲酸水溶液和0.1%(v/v)甲酸乙腈溶液-0.1%(v/v)甲酸水溶液作为流动相时的色谱分离效果。结果表明,以甲醇-水为流动相时,大环内酯类抗生素未出峰,在其他的流动相条件下,各类抗生素均有出峰。此外,四环素类抗生素的响应在含醋酸铵的流动相条件下有所降低,青霉素的响应在0.1%(v/v)甲酸乙腈溶液作为有机相的条件下较0.1%(v/v)甲酸甲醇溶液低了2个数量级。综合考虑,选取0.1%(v/v)甲酸甲醇溶液-0.1%(v/v)甲酸水溶液作为流动相。

此外,纯甲醇作为进样溶剂时,溶剂效应显著,阿莫西林未出峰。当有机溶剂的比例降低时,各类抗生素的峰形及响应均有所改善,各类抗生素的响应在以10%(v/v)甲醇水溶液作为进样溶剂时较50%(v/v)甲醇水溶液高2~10倍。因此选取10%甲醇水溶液作为进样溶剂。30种抗生素(100 μg/L)的总离子流色谱图见图1。

**图1:**
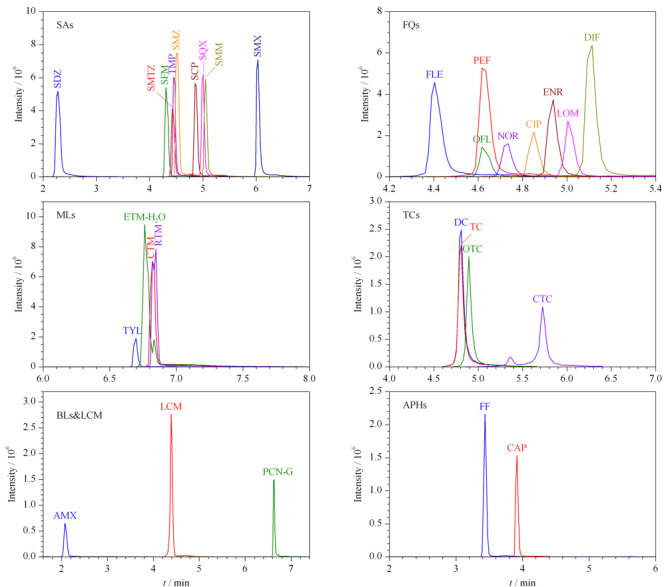
30种抗生素(100 μg/L)的提取离子流色谱图

### 2.2 提取条件的优化

采用振荡加超声的方式对土壤样品中的抗生素进行提取,考察不同种类(甲醇-Na_2_EDTA-McIlvaine缓冲液(1∶1, v/v)和乙腈-Na_2_EDTA-McIlvaine缓冲液(1∶1, v/v))和不同体积(5、7.5和10 mL)的萃取剂对目标化合物提取效果的影响。结果表明,与甲醇-Na_2_EDTA-McIlvaine缓冲液相比,乙腈-Na_2_EDTA-McIlvaine缓冲液对氟喹诺酮类抗生素有更好的提取效果,这可能是因为氟喹诺酮类抗生素极性较大(log *K*_ow_: -1.03~0.89)。以乙腈-Na_2_EDTA-McIlvaine缓冲液作为萃取剂,萃取剂的体积由5 mL提升至10 mL时,氟喹诺酮类抗生素的回收率由15.0%~44.0%提升至48.0%~84.0%,部分磺胺类和大环内酯类抗生素的回收率也有10.0%~20.0%的提升。每次使用10 mL萃取剂对土壤样品进行提取,经3次提取后,第4次的提取液中未检测到目标化合物。因此,最终选择采用乙腈-Na_2_EDTA-McIlvaine缓冲液(1∶1, v/v)对土壤样品提取3次,每次10 mL。

### 2.3 净化条件的优化

2.3.1 萃取液pH值的选择

未经调节的萃取液(乙腈-Na_2_EDTA-McIlvaine缓冲液(1∶1, v/v))pH约为4.0,在此条件下进行固相萃取,*β*-内酰胺类抗生素中的阿莫西林回收率仅为10.3%,可能是酸性条件下阿莫西林易降解^[15]^。探究中性到碱性的3个pH条件(pH 7.0、8.0、9.0)对各类抗生素回收率的影响。

结果表明,萃取液pH的变化对林可霉素、氟喹诺酮类和酰胺醇类抗生素的回收率影响不大,而pH 8.0为磺胺类、大环内酯类、四环素类及*β*-内酰胺类抗生素的最佳萃取液pH值,该条件下4类抗生素的回收率范围在30.0%~132%(见图2)。最终确定萃取液pH调节为8.0。

**图2:**
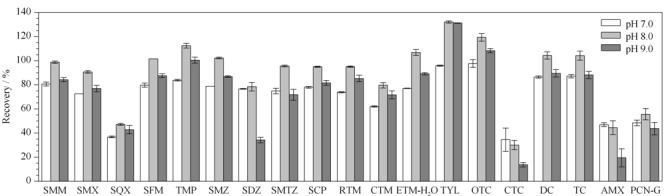
萃取液的pH值对磺胺类、大环内酯类、四环素类及*β*-内酰胺类抗生素回收率的影响(*n*=3)

2.3.2 淋洗液有机溶剂比例的选择

为了保留目标化合物并最大化地去除杂质,选取10 mL超纯水、5 mL超纯水+5 mL 5%(v/v)甲醇水溶液和5 mL超纯水+5 mL 10%(v/v)甲醇水溶液作为淋洗液,以确定最佳的淋洗液有机溶剂的比例。结果表明,随着淋洗液中有机溶剂比例的增加,大多数抗生素回收率无明显变化,但是磺胺嘧啶和阿莫西林的回收率分别由76.6%和47.0%降低至19.5%和4.10%。甲醇是磺胺嘧啶和阿莫西林最常用的洗脱溶剂^[6,14]^,磺胺嘧啶具有一定的亲水性(log *K*_ow_: -0.09),阿莫西林是一种强极性化合物,这可能导致它们弱吸附于反相固相萃取小柱填料,并且在含有少量甲醇的淋洗液中即可被洗脱。此结果与目标化合物在反相BEH-C18色谱柱上的洗脱情况一致,磺胺嘧啶和阿莫西林的保留时间分别为2.16 min和1.86 min,是所有目标化合物中出峰时间最早的两种抗生素。因此,确定将10 mL超纯水作为固相萃取的淋洗液。这与Zhou等^[14]^的研究结果一致。

2.3.3 洗脱液种类及体积的选择

甲醇是最常被报道的抗生素的洗脱溶剂^[14,16]^,由于目标抗生素的极性普遍较大(log *K*_ow_: -4.10~4.34),分别选取甲醇、乙腈和甲醇-乙腈(1∶1, v/v)混合溶液作为洗脱液,以考察不同洗脱液对抗生素洗脱效果的影响。结果显示,甲醇-乙腈(1∶1, v/v)混合溶液作为洗脱液时,各类抗生素的洗脱效果最佳,回收率范围为34.5%~177%。

考察不同体积的洗脱液(6、10、12 mL)对抗生素洗脱效果的影响。结果显示,对于酰胺醇类和部分磺胺类、氟喹诺酮类抗生素,6 mL洗脱液达不到理想的洗脱效果,而各类抗生素的回收率在用12 mL洗脱液洗脱的情况下较10 mL并没有得到明显提高。由此可见,10 mL洗脱液足以将固相萃取柱上的抗生素洗脱完全。更大的体积不仅浪费试剂,增加前处理时间,还可能带来基质效应。因此,最终确定洗脱液体积为10 mL。

### 2.4 方法学评价

2.4.1 线性范围与方法检出限

经UHPLC-MS/MS检测系列混合标准溶液后,以抗生素的质量浓度为横坐标(*x*, μg/L),峰面积为纵坐标(*y*),绘制标准曲线。结果表明,所有抗生素的标准曲线的相关系数(*r*)均大于0.99(见表2)。分别以3倍和10倍信噪比计算方法检出限和定量限,30种抗生素的方法检出限和定量限分别为0.013~1.21和0.043~4.04 μg/kg。在低(2 μg/L)、中(20 μg/L)和高浓度(100 μg/L)条件下测定日内和日间精密度,30种抗生素的日内和日间精密度分别为0.238%~14.1%和0.507%~14.5%,均小于15%,证实了方法的重复性。

**表2:** 30种抗生素的方法检出限、定量限、线性范围及在3个浓度水平下的日内和日间精密度

Compound	LOD/ (μg/kg)	LOQ/ (μg/kg)	Linear range/ (μg/L)	*r*	RSDs (intra-day)/%				RSDs (inter-day)/%		
					2 μg/L	20 μg/L	100 μg/L		2 μg/L	20 μg/L	100 μg/L
SMM	0.116	0.388	0.01-100	0.997	9.63	2.08	0.619		6.33	5.87	2.08
SMX	0.0459	0.153	0.01-200	0.999	8.81	3.23	1.36		3.26	3.52	2.16
SQX	0.0278	0.0926	0.01-100	0.999	11.3	2.61	1.38		4.52	3.43	1.53
SFM	0.107	0.356	0.01-200	0.999	1.24	1.42	2.59		5.86	4.70	4.49
TMP	0.0388	0.129	0.01-100	0.999	8.68	6.95	1.09		3.95	2.65	2.21
SMZ	0.0284	0.0946	0.01-200	0.999	1.07	1.88	0.613		4.38	1.04	4.06
SDZ	0.0130	0.0433	0.01-200	0.999	5.69	0.730	2.56		2.63	0.507	2.97
SMTZ	0.0325	0.108	0.02-100	1.000	5.48	0.274	3.22		8.68	6.11	12.2
SCP	0.0612	0.204	0.01-100	0.996	11.8	3.11	2.40		6.08	6.00	3.04
RTM	0.0673	0.224	0.01-100	0.999	1.41	6.96	3.83		3.86	10.1	1.19
CTM	0.0492	0.164	0.01-100	0.999	4.76	2.84	3.25		13.1	10.7	1.74
ETM-H_2_O	0.0313	0.104	0.02-100	0.999	5.98	2.42	4.44		13.1	4.53	4.97
TYL	0.104	0.347	0.5-200	1.000	3.36	3.58	0.515		14.0	13.8	14.2
CIP	0.623	2.08	2-200	0.998	10.1	3.13	13.7		7.17	13.9	6.54
NOR	0.619	2.06	2-200	0.997	1.36	4.43	12.1		2.86	9.70	12.9
OFL	0.112	0.373	1-200	0.992	9.05	3.38	6.28		9.97	12.8	5.54
ENR	0.104	0.348	1-200	0.999	11.6	3.04	8.07		12.0	8.67	1.15
DIF	0.147	0.492	0.02-200	0.998	10.5	3.11	4.19		9.96	3.37	3.62
PEF	0.197	0.658	0.2-200	0.998	6.00	2.22	12.4		10.0	3.70	4.64
FLE	0.0513	0.171	0.1-100	0.999	9.41	5.31	1.36		3.55	14.0	7.65
LOM	0.129	0.429	0.2-100	0.998	11.9	4.38	3.58		11.4	5.12	13.9
OTC	0.122	0.407	0.5-200	0.995	4.89	4.03	8.99		8.81	3.79	8.31
CTC	1.21	4.04	0.02-200	0.998	11.5	5.23	2.51		13.7	7.91	6.42
DC	0.168	0.560	0.1-200	0.997	14.1	1.82	5.05		13.3	1.60	14.5
TC	0.209	0.698	0.02-200	0.997	11.3	4.66	3.81		7.28	2.85	14.3
AMX	0.119	0.395	0.5-200	0.999	0.964	1.55	8.62		2.96	14.4	13.5
PCN-G	0.134	0.446	0.2-200	1.000	10.4	1.54	4.59		14.3	11.0	11.7
LCM	0.0251	0.0836	0.02-200	0.998	11.4	4.74	0.476		11.4	8.93	6.44
CAP	1.06	3.55	0.01-200	0.998	2.67	2.84	0.238		10.8	12.5	8.81
FF	0.166	0.553	0.01-200	0.998	2.42	1.39	4.90		13.1	9.88	7.60

2.4.2 回收率与基质效应

称量0.25 g土壤样品,添加抗生素标准品,使得加标水平分别为20、100和200 μg/kg,每个浓度水平设4组平行。结果见表3,可以看出,仅有个别目标化合物(磺胺喹噁啉、阿莫西林和青霉素)的回收率为27.1%~31.5%,其他27种抗生素的加标回收率均在44.8%~164%之间,并且30种抗生素的相对标准偏差为0.700%~14.8%,均<15%,说明该分析方法性能良好。

**表3:** 30种抗生素在土壤样品中的加标回收率和基质效应

Compound	Recoveries (RSDs)/% (*n*=4)			Matrix effect/%	Compound	Recoveries (RSDs)/% (*n*=4)			Matrix effect/%
	20 μg/kg	100 μg/kg	200 μg/kg			20 μg/kg	100 μg/kg	200 μg/kg	
SMM	79.0 (3.02)	53.0 (12.0)	51.4 (5.69)	48.0-56.2	OFL	126.0 (2.67)	87.1 (14.8)	75.2 (10.2)	73.9-110
SMX	94.4 (3.71)	72.3 (12.6)	82.1 (11.3)	51.5-65.3	ENR	78.0 (14.4)	77.0 (12.9)	70.7 (6.53)	68.4-110
SQX	45.2 (4.39)	31.5 (13.7)	27.1 (7.26)	27.7-34.2	DIF	109.0 (2.57)	78.8 (12.0)	63.3 (5.48)	61.4-73.7
SFM	111.0 (8.60)	76.3 (8.21)	70.9 (10.1)	67.8-78.1	PEF	128.0 (5.61)	125.0 (12.5)	88.0 (11.3)	94.6-138
TMP	123.0 (2.77)	84.4 (13.1)	70.6 (10.0)	68.0-78.0	FLE	90.2 (6.56)	51.9 (14.5)	44.8 (10.0)	45.5-71.7
SMZ	101.0 (5.05)	66.4 (8.97)	68.5 (7.88)	62.5-75.6	LOM	71.4 (13.5)	69.5 (10.3)	55.8 (12.7)	64.6-78.0
SDZ	94.2 (14.4)	87.6 (14.8)	75.5 (9.10)	71.5-86.7	OTC	164.0 (6.40)	121.0 (11.0)	87.8 (4.86)	73.3-116
SMTZ	101.0 (6.75)	57.4 (11.3)	53.7 (11.7)	63.1-72.9	CTC	136.0 (15.2)	122.0 (17.3)	143.0 (7.58)	48.3-67.3
SCP	90.5 (2.51)	57.9 (10.1)	55.3 (8.06)	56.7-65.5	DC	138.0 (6.29)	74.5 (7.50)	70.7 (5.52)	69.6-115
RTM	89.5 (9.01)	73.7 (8.46)	60.9 (3.43)	68.3-133	TC	146.0 (11.5)	79.2 (12.8)	69.5 (8.45)	73.8-119
CTM	54.5 (10.1)	53.1 (10.3)	45.3 (3.64)	56.8-81.1	AMX	29.6 (6.11)	45.1 (5.39)	43.0 (9.40)	66.5-91.0
ETM-H_2_O	57.8 (12.8)	57.6 (13.4)	48.9 (0.700)	53.7-70.9	PCN-G	78.5 (3.31)	29.1 (2.76)	30.6 (8.90)	38.3-40.0
TYL	67.3 (8.16)	75.5 (11.0)	70.8 (3.99)	91.5-115	LCM	70.9 (7.37)	74.9 (4.17)	55.3 (5.69)	74.5-88.4
CIP	45.8 (11.9)	82.9 (10.9)	69.0 (6.11)	91.4-134	FF	96.5 (5.94)	59.9 (11.7)	46.9 (8.50)	44.4-52.4
NOR	64.2 (5.17)	107.0 (11.9)	89.1 (7.73)	137.0-172	CAP	146.0 (8.11)	95.3 (13.5)	80.9 (5.79)	69.4-81.6

磺胺喹噁啉、阿莫西林和青霉素的化学结构和理化性质与其他27种抗生素相比有一定差异。磺胺喹噁啉的log*K*_ow_为1.68,亲水性远小于其他7种磺胺类抗生素(log *K*_ow_: -0.09~0.91)。阿莫西林和青霉素属于*β*-内酰胺类抗生素,在*β*-内酰胺环的环张力和羰基碳的亲核能力作用下,这类抗生素具有高度的化学反应活性^[17]^。由于理化性质的差异,在30种抗生素同时分析测定的过程中,这3种抗生素可能存在一定的过程损失或较强的基质抑制作用,从而导致回收率偏低。

在20、100和200 μg/kg的添加浓度下,比较基质提取液和纯溶剂中30种抗生素的响应值,以评估基质效应(ME),结果见表3。ME<100%为基质抑制作用,ME>100%为基质增强作用。ME<50%或>150%为强基质作用,50%≤ME≤80%或120%≤ME≤150%为中等基质作用,80%<ME<120%为弱基质作用^[18]^。磺胺喹噁啉和青霉素表现为强基质抑制作用(27.7%≤ME≤40.0%),这可能导致了其较低的回收率(27.1%~31.5%)。其他28种抗生素的ME范围为44.4%~172%,部分抗生素的回收率可能受其影响。如脱水红霉素的回收率范围为48.9%~57.8%,这可能是由于其中等的基质抑制作用(53.7%≤ME≤70.9%)。

不同地区的土壤性质各异,针对每个地区的土壤样品制备基质匹配标准曲线的工作量巨大,不能满足简单快速进行定量分析的要求;此外,多种抗生素在土壤中的检出率基本为100%,难以获得空白基质制备基质匹配标准曲线。因此,本工作使用溶剂标准曲线进行定量。

2.4.3 方法学比较

与其他已有文献数据比较(见表4),该方法的萃取溶剂用量少,且涉及的抗生素种类广、数量多,能用于土壤样品中痕量水平7类30种抗生素的同时分析检测。其中磺胺类、氟喹诺酮类、四环素类、大环内酯类、林可酰胺类和酰胺醇类共27种抗生素的加标回收率范围为44.8%~164%。本研究中磺胺喹噁啉(27.1%~45.2%)、阿莫西林(29.6%~45.1%)和青霉素(29.1%~78.5%)的回收率较低,与其他研究^[6,19,20]^报道一致。如Guo等^[20]^采用QuEChERS-HPLC-MS/MS测定粪便中25种抗生素,其中磺胺喹噁啉的回收率最低(55.7%~56.8%); Cha等^[19]^利用固相萃取-HPLC-MS/MS检测粪便中*β*-内酰胺类抗生素,阿莫西林的回收率只有15%左右。

**表4:** 本文方法与其他文献方法的比较

Matrix	Category	*N* ^3)^	Recoveries/%	LOQs/ (μg/kg)	Extraction solvent	Purification and detection method	Reference
Soil	SAs, FQs, TCs, MLs, BLs, LAs^1)^, APHs	30	27.1-164	0.0430-4.04	ACN/Na_2_EDTA-McIlvaine (1∶1, v/v, 10 mL×3)	HLB-UHPLC-MS/MS	this work
Soil	MLs, FQs, LAs	9	65.2-106	0.800-9.67	ACN/Na_2_EDTA-H_3_PO_4_ (1∶1, v/v, 20 mL×2)	SAX-HLB-UHPLC-MS/MS	[10]
Soil	SAs, BLs, APHs	13	78.5-107	1.33-3.33	ACN/Na_2_EDTA-McIlvaine (1∶1, v/v, 20 mL×3)	HLB-HPCE	[11]
Soil	SAs, MLs	20	61.4-119	2.00-5.00	QuEChERS	HPLC-MS/MS	[12]
Soil	SAs, TCs	6	40-114	0.600-2.30	Na_2_EDTA-McIlvaine (20 mL)	SPE-HPLC-MS/MS	[21]
Soil	SAs, FQs, MLs	22	53.3-112	0.00670-0.017	ACN/Na_2_EDTA-McIlvaine (2/1, v/v, 15 mL×3)	HLB-HPLC-MS/MS	[22]
Manure	BLs	5	15.0-87.1^4)^	1.13-6.20^5)^		SPE-HPLC-MS/MS	[19]
Manure	SAs, FQs, TCs, MLs, LAs, PMs^2)^	25	61.4-106 (SQX: 55.7-56.8)	0.05-5.91	QuEChERS	HPLC-MS/MS	[20]
Waste-water	SAs, FQs, MLs, BLs, LAs, APHs	11	20-180	0.01-0.02^6)^		HLB-HPLC-MS/MS	[6]

1) LAs: lincosamides; 2) PMs: pleuromutilins; 3) *N*: number of target analytes; 4) recoveries of five *β*-lactams (amoxicillin and ampicillin: ~15%); 5) LOQs of three *β*-lactams except for amoxicillin and ampicillin; 6) μg/L.

2.4.4 实际土壤样品的测定

分别采集来自郑州、河池、聊城、衡阳、莱州和白银6个地区的6个农田表层土壤样品,使用建立的方法对土壤样品中30种抗生素残留进行分析,统计结果见表5,具体的浓度信息见图3。共检出30种抗生素中的17种,在每个样品中的总含量为73.4~184 μg/kg,其余抗生素均未检出。罗红霉素、克拉霉素、环丙沙星、诺氟沙星、氧氟沙星、恩诺沙星、氟罗沙星、洛美沙星、土霉素、多西环素、四环素和青霉素的检出率均为100%,其中环丙沙星和诺氟沙星是每个土壤样品中含量最高的两种抗生素(见图3),它们的含量分别是13.7~32.1和15.6~43.6 μg/kg,罗红霉素、克拉霉素、氧氟沙星、恩诺沙星、氟罗沙星、洛美沙星、土霉素、多西环素、四环素和青霉素的含量分别是0.824~1.41、0.255~0.496、5.83~16.1、4.37~11.1、0.503~3.49、2.27~9.80、3.14~14.0、3.63~14.3、3.45~14.4和2.74~11.1 μg/kg。磺胺喹噁啉、甲氧苄啶、泰乐菌素、双氟沙星和培氟沙星分别在1、3、4、4和2个土样中被检出,它们的含量分别为nd~0.238、nd~0.235、nd~1.23、nd~7.75和nd~8.51 μg/kg。主要抗生素的检出率和浓度水平情况与之前研究报道的结果^[21,22,23,24,25,26,27,28,29]^基本一致。如Sun等^[27]^对中国长三角地区农田土壤中13 种抗生素残留水平(磺胺类、四环素类和氟喹诺酮类)进行了分析,其中环丙沙星含量为未检出~1030 μg/kg,其均值在所有抗生素中最高,为27.7 μg/kg; Gao等^[22]^测定了北京和上海城市土壤中的22种抗生素(磺胺类、氟喹诺酮类和大环内酯类),其中诺氟沙星在所有样本中检出率最高(98%),含量为未检出~2160 μg/kg,均值为94.6 μg/kg。这说明环丙沙星和诺氟沙星广泛而大量存在于我国土壤环境中。

**表5:** 6个实际土壤样品中30种抗生素的含量统计

Compound	GM/ (μg/kg)	Min/ (μg/kg)	Max/ (μg/kg)	DF/%	Compound	GM/ (μg/kg)	Min/ (μg/kg)	Max/ (μg/kg)	DF/%
SMM	0.274	nd	nd	0	OFL	8.07	5.83	16.1	100
SMX	0.108	nd	nd	0	ENR	6.41	4.37	11.1	100
SQX	0.0812	nd	0.238	16.7	DIF	2.02	nd	7.75	83.3
SFM	0.252	nd	nd	0	PEF	0.995	nd	8.51	33.3
TMP	0.125	nd	0.235	50.0	FLE	0.952	0.503	3.49	100
SMZ	0.0669	nd	nd	0	LOM	3.96	2.27	9.80	100
SDZ	0.0306	nd	nd	0	OTC	6.23	3.14	14.0	100
SMTZ	0.0767	nd	nd	0	CTC	2.86	nd	nd	0
SCP	0.144	nd	nd	0	DC	6.14	3.63	14.3	100
RTM	1.00	0.824	1.41	100	TC	5.79	3.45	14.4	100
CTM	0.326	0.255	0.496	100	AMX	0.279	nd	nd	0
ETM-H_2_O	0.0738	nd	nd	0	PCN-G	4.57	2.74	11.1	100
TYL	0.658	nd	1.23	83.3	LCM	0.0591	nd	nd	0
CIP	19.5	13.7	32.1	100	FF	0.391	nd	nd	0
NOR	24.2	15.6	43.6	100	CAP	2.51	nd	nd	0

GM: geometric mean; Min: minimum; Max: maximum; DF: detection frequency; nd: not detected.

**图3:**
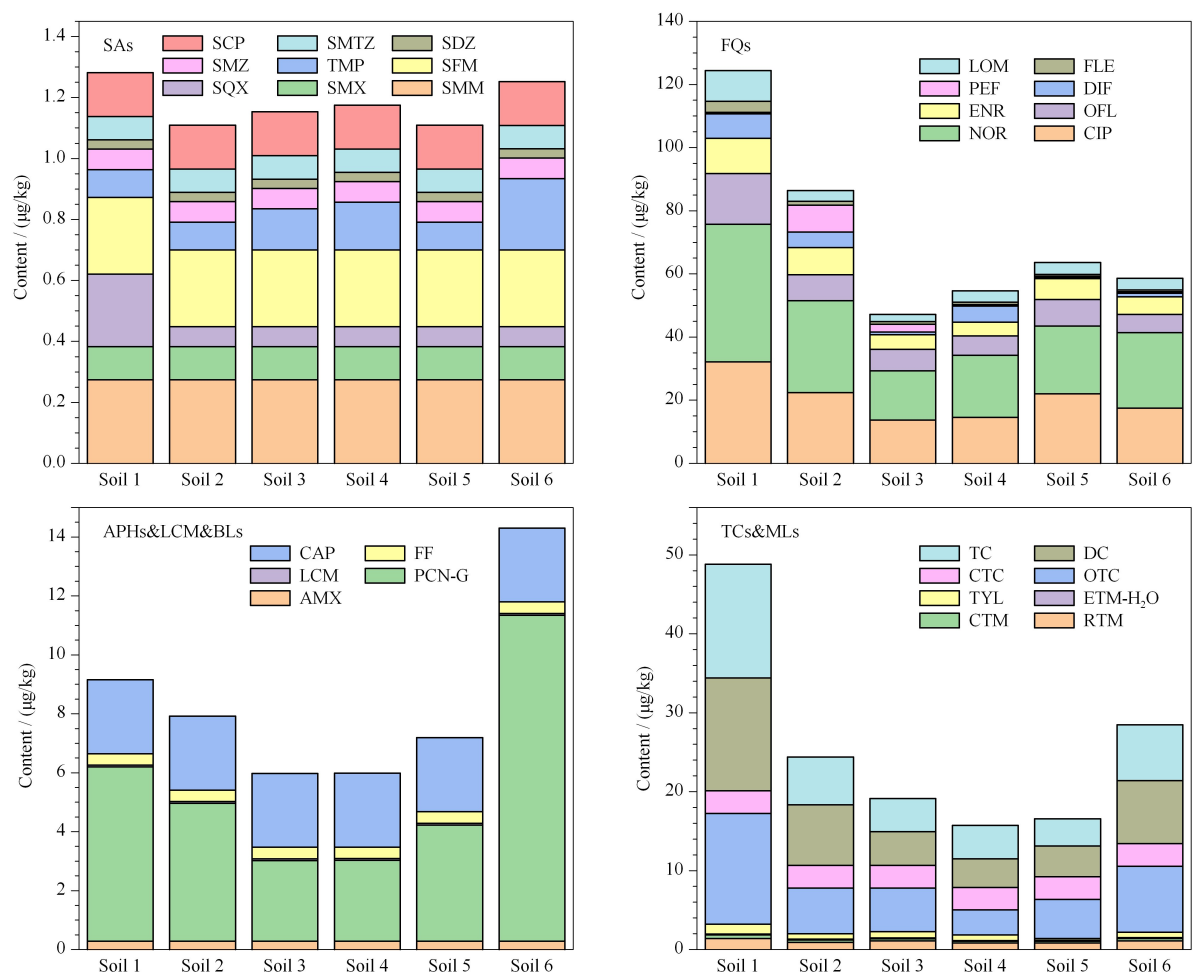
6个实际土壤样品中30种抗生素的含量及分布情况

## 3 结论

本研究建立了固相萃取-超高效液相色谱-串联质谱同时测定土壤中痕量水平的7类30种抗生素的方法。该方法的线性范围、检出限、回收率及精密度等方法学指标均可满足土壤中抗生素的定性定量分析要求,且方法简单快速、溶剂用量少、抗生素检测的种类广、数量多,可望为土壤中抗生素的监测和风险评估提供方法学基础。

## References

[1] Zhang Q Q, Ying G G, Pan C G, et al. Environ Sci Technol, 2015,49(11):6772 25961663 10.1021/acs.est.5b00729

[2] Ezzariai A, Hafidi M, Khadra A, et al. J Hazard Mater, 2018,359:465 30071464 10.1016/j.jhazmat.2018.07.092

[3] Seifrtova M, Novakova L, Lino C, et al. Anal Chim Acta, 2009,649(2):158 19699391 10.1016/j.aca.2009.07.031

[4] Wang Z, Shi Z, Xi C, et al. Food Addit Contam A, 2017,34(12):2144 10.1080/19440049.2017.1382724 28934031

[5] Ministry of Agriculture and Rural Affairs. No. 194 Bulletin of the Ministry of Agriculture and Rural Affairs of the People’s Republic of China. (2019-07-10) [2021-05-11]. http://www.xmsyj.moa.gov.cn/zcjd/201907/t20190710_6320678.htmhttp://www.xmsyj.moa.gov.cn/zcjd/201907/t20190710_6320678.htm

[6] Brown K D, Kulis J, Thomson B, et al. Sci Total Environ, 2006,366(2/3):772 16313947 10.1016/j.scitotenv.2005.10.007

[7] Galvidis I, Lapa G, Burkin M. Anal Biochem, 2015,468:75 25256165 10.1016/j.ab.2014.09.009

[8] Legrand T, Vodovar D, Tournier N, et al. Antimicrob Agents Chemother, 2016,60(8):4734 27216076 10.1128/AAC.00176-16 PMC4958196

[9] Merola G, Martini E, Tomassetti M, et al. J Pharm Biomed Anal, 2015,106:186 25178531 10.1016/j.jpba.2014.08.005

[10] Li X J, Yu H, Gan P S. Journal of Environmental Hygiene, 2016,6(4):296

[11] Li X H, Miao J J, Kang K, et al. Physical Testing and Chemical Analysis Part B: Chemical Analysis, 2019,55(7):769

[12] Meng M H, He Z Y, Xu Y P, et al. Journal of Agro-Environment Science, 2017,36(8):1672

[13] Wang R, Feng F, Chai Y, et al. Sci Total Environ, 2019,660:1542 30743946 10.1016/j.scitotenv.2019.01.127

[14] Zhou L J, Ying G G, Liu S, et al. J Chromatogr A, 2012,1244:123 22625208 10.1016/j.chroma.2012.04.076

[15] Khuroo A H, Monif T, Verma P R P, , et al. J Chromatogr Sci, 2008,46(10):854 19007491 10.1093/chromsci/46.10.854

[16] Zhang M, Liu Y S, Zhao J L, et al. Sci Total Environ, 2018,639:1421 29929305 10.1016/j.scitotenv.2018.05.230

[17] Cha J M, Yang S, Carlson K H. J Chromatogr A, 2006,1115(1/2):46 16595135 10.1016/j.chroma.2006.02.086

[18] Martinez Pierna A B, Polo Lopez M I, Fernandez Ibanez P, et al. J Chromatogr A, 2018,1534:10 29277255 10.1016/j.chroma.2017.12.037

[19] Cha J, Carlson K H. Sci Total Environ, 2018,640:1346 30021301 10.1016/j.scitotenv.2018.05.391

[20] Guo C, Wang M, Xiao H, et al. J Chromatogr B, 2016,1027:110 10.1016/j.jchromb.2016.05.034 27276651

[21] Awad Y M, Kim S C, Abd El Azeem S A M, , et al. Environ Earth Sci, 2014,71(3):1433

[22] Gao L, Shi Y, Li W, et al. Environ Sci Pollut Res, 2015,22(15):11360 10.1007/s11356-015-4230-3 25804657

[23] Guo T, Lou C, Zhai W, et al. Sci Total Environ, 2018,635:995 29710621 10.1016/j.scitotenv.2018.04.194

[24] Hou J, Wan W, Mao D, et al. Environ Sci Pollut Res, 2015,22(6):4545 10.1007/s11356-014-3632-y 25318415

[25] Huang X, Liu C, Li K, et al. Environ Sci Pollut Res, 2013,20(12):9066 10.1007/s11356-013-1905-5 23812733

[26] Li C, Chen J, Wang J, et al. Sci Total Environ, 2015,521:101 25828417 10.1016/j.scitotenv.2015.03.070

[27] Sun J, Zeng Q, Tsang D C W, , et al. Chemosphere, 2017,189:301 28942256 10.1016/j.chemosphere.2017.09.040

[28] Xiang L, Wu X L, Jiang Y N, et al. Environ Sci Pollut Res, 2016,23(14):13984 10.1007/s11356-016-6493-8 27040546

[29] Zeng Q, Sun J, Zhu L. Chemosphere, 2019,224:900 30986896 10.1016/j.chemosphere.2019.02.167

